# Mendelian randomization to evaluate the effect of plasma vitamin C levels on the risk of Alzheimer’s disease

**DOI:** 10.1186/s12263-021-00700-9

**Published:** 2021-10-29

**Authors:** Haijie Liu, Yan Zhang, Yang Hu, Haihua Zhang, Tao Wang, Zhifa Han, Shan Gao, Longcai Wang, Guiyou Liu

**Affiliations:** 1 Department of Neurology, Xuanwu Hospital, Capital Medical University, Beijing, 100053 China; 2 Department of Pathology, The Affiliated Hospital of Weifang Medical University, Weifang, 261053 China; 3 School of Life Science and Technology, Harbin Institute of Technology, Harbin, 150080 China; 4 Beijing Institute of Brain Disorders, Laboratory of Brain Disorders, Ministry of Science and Technology, Collaborative Innovation Center for Brain Disorders, Capital Medical University, Beijing, 100069 China; 5 Academy for Advanced Interdisciplinary Studies, Peking University, Beijing, 100871 China; 6 Chinese Institute for Brain Research, Beijing, 102206 China; 7 School of Medicine, School of Pharmaceutical Sciences, THU-PKU Center for Life Sciences, Tsinghua University, Beijing, 100091 China; 8 Department of Anesthesiology, The Affiliated Hospital of Weifang Medical University, Weifang, 261053 China; 9 National Engineering Laboratory of Internet Medical Diagnosis and Treatment Technology, Xuanwu Hospital, Capital Medical University, Beijing, 100053 China; 10 Beijing Key Laboratory of Hypoxia Translational Medicine, Xuanwu Hospital, Capital Medical University, Beijing, 100053 China

**Keywords:** Alzheimer’s disease, Vitamin C, Genome-wide association study, Mendelian randomization, Inverse-variance weighted

## Abstract

**Objective:**

Until now, observational studies have explored the impact of vitamin C intake on Alzheimer’s disease (AD) risk, however, reported ambiguous findings. To develop effective therapies or prevention, the causal link between vitamin C levels and AD should be established.

**Methods:**

Here, we selected 11 plasma vitamin C genetic variants from a large-scale plasma vitamin C GWAS dataset (*N* = 52,018) as the potential instrumental variables. We extracted their corresponding summary statistics from large-scale IGAP clinically diagnosed AD GWAS dataset (*N* = 63,926) and UK Biobank AD proxy phenotype GWAS dataset (*N* = 314,278), as well as two UK Biobank subgroups including the maternal AD group (27,696 cases of maternal AD and 260,980 controls) and paternal AD group (14,338 cases of paternal AD and 245,941 controls). We then performed a Mendelian randomization (MR) study to evaluate the causal association between plasma vitamin C levels and the risk of AD and AD proxy phenotype. Meanwhile, we further verified these findings using a large-scale cognitive performance GWAS dataset (*N* = 257,841).

**Results:**

In IGAP, we found no significant causal association between plasma vitamin C levels and the risk of AD. In UK Biobank, we found that per 1 *SD* increase in plasma vitamin C levels (about 20.2 μmol/l) was significantly associated with the reduced risk of AD proxy phenotype (*OR* = 0.93, *95% CI* 0.88–0.98, *P* = 7.00E−03). A subgroup MR analysis in UK Biobank indicated that per 1 *SD* increase in plasma vitamin C levels could significantly reduce the risk of AD proxy phenotype in the maternal AD group (*OR* = 0.89, *95% CI* 0.84–0.94, *P* = 7.29E−05), but not in the paternal AD group (*OR* = 1.02, *95% CI* 0.92–1.12, *P* = 7.59E−01). The leave-one-out permutation further showed that the *SLC23A1* rs33972313 variant largely changed the precision of the overall MR estimates in all these four GWAS datasets. Meanwhile, we did not observe any significant causal effect of plasma vitamin C levels on the cognitive performance.

**Conclusion:**

We demonstrated that there may be no causal association between plasma vitamin C levels and the risk of AD in people of European descent. The insistent findings in clinically diagnosed AD and AD proxy phenotype may be caused by the phenotypic heterogeneity.

**Supplementary Information:**

The online version contains supplementary material available at 10.1186/s12263-021-00700-9.

## Introduction

Alzheimer’s disease (AD) is the most common neurodegenerative disease, which is characterized by **extracellular deposition** of amyloid plaques mainly composed of β-amyloid protein (Aβ) [1]. Meanwhile, it is reported that oxidative stress is involved in the pathogenesis of AD [2, 3]. The soluble oligomeric forms of Aβ could induce neurotoxicity in neuronal cell cultures, which is triggered by reactive oxygen species [2, 4]. Vitamin C is an important water-soluble antioxidant, which could neutralize reactive oxygen species and reduce the oxidative stress [3]. Evidence from animal models of AD highlighted the protective role of vitamin C in AD [5]. Kook and colleagues found that the high-dose supplementation of vitamin C could reduce amyloid plaque burden in the cortex and hippocampus in 5 familial AD mutation (5XFAD) mice and further ameliorate the blood–brain barrier disruption and mitochondrial alteration [5].

Until now, observational studies have investigated the role of vitamin C intake (both diet and supplements) in AD, however, reported ambiguous findings [6–10]. The Rotterdam Study, a population-based, prospective cohort study in the Netherlands, showed that high intake of vitamin C was associated with reduced AD risk with rate ratio = 0.82 and 95% confidence interval (*CI*) 0.68–0.99 corresponding per 1 *SD* increase in intake [6]. The Rush Memory and Aging Project (MAP) indicated that highest vs. lowest quartile intakes of vitamin C were associated with reduced AD risk (hazard ratio = 0.64, *95% CI* 0.45–0.92) [10]. The Washington Heights-Inwood Columbia Aging Project (WHICAP) showed neither dietary nor supplemental vitamin C was associated with the decreased risk of AD [7]. The Cache County Study indicated no evidence of a protective effect with the use of vitamin C supplements to reduce the prevalence and incidence of AD [8]. The Chicago Health and Aging Project (CHAP) found that total vitamin C intake from foods and supplements or vitamin C intake from foods alone was not significantly associated with the risk of AD [9].

In order to develop effective therapies or prevention, the causal link between vitamin C levels and AD should be established. Hence, it is necessary to improve the causal inference through other study designs and overcome the methodological limitations of observational studies [11]. Interestingly, Mendelian randomization (MR) design has been widely used to determine the causal inferences in AD, such as vitamin D levels and AD, vitamin E levels and AD, and vitamin B12 levels and AD [11–16]. Meanwhile, Williams and colleagues recently conducted a MR analysis to evaluate the causal association of four circulating antioxidants including circulating ascorbate (vitamin C), β-carotene, retinol (vitamin A), and urate with the risk of AD [17]. Williams and colleagues found that increased circulating levels of ascorbate, β-carotene, retinol, or urate could not reduce the risk of AD [17]. They suggested that MR studies should further verify these findings using larger AD case–control samples and more additional variants as the instrument [17]. Until now, large-scale AD or AD proxy phenotype GWAS datasets and newly identified vitamin C genetic variants are publicly available [18–20]. Here, we performed an updated two-sample MR study to investigate the causal association of plasma vitamin C levels with AD and AD proxy phenotype using large-scale plasma vitamin C genome-wide association study (GWAS) dataset, clinically diagnosed AD GWAS dataset from IGAP, and three AD proxy phenotype GWAS datasets from UK Biobank [18–20]. Meanwhile, we further verified these findings using a large-scale cognitive performance GWAS dataset [21].

## Materials and methods

### Study design

**Fig. 1:**
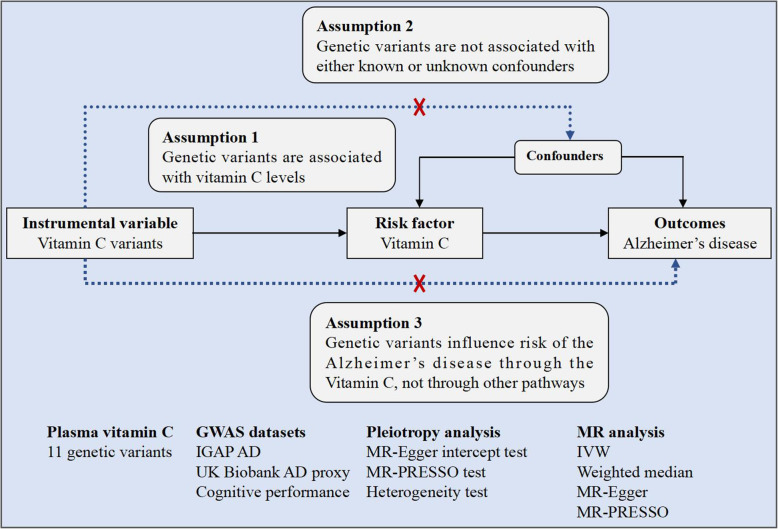
The flow chart about the MR study design

Our study design is a two-sample MR study, which is based on the large-scale publicly available GWAS summary datasets in plasma vitamin C, AD, AD proxy phenotype, and cognitive performance [18–21]. All participants have given informed consent in all these corresponding original studies [18–21]. In general, MR must meet three principal assumptions [22–26]. First, genetic variants should be significantly associated with plasma vitamin C levels (*P* < 5.00E−08). Second, plasma vitamin C genetic variants should not be associated with confounders. Third, plasma vitamin C genetic variants should affect the risk of AD only through plasma vitamin C levels. Figure 1 provides a flow chart about our MR study design.

### Vitamin C GWAS dataset

The vitamin C GWAS dataset is from a recent large-scale GWAS meta-analysis of plasma vitamin C in 52,018 individuals of European ancestry [18]. In brief, these 52,018 samples are from (1) the Fenland study, an ongoing, population-based cohort study (*n* = 10,771) in Cambridgeshire, UK; (2) European Prospective Investigation into Cancer and Nutrition (EPIC)-InterAct study, a case–cohort study (*n* = 16,841) from eight European countries (France, Italy, Spain, UK, Netherlands, Germany, Sweden, and Denmark); (3) EPIC-Norfolk study, an ongoing UK-based prospective cohort study (*n* = 16,756, excluding overlapped samples with EPIC-InterAct); and (4) EPIC-CVD study, a case–cohort study (*n* = 7650, excluding overlapped samples with EPIC-InterAct or EPIC-Norfolk) [18]. The vitamin C GWAS has adjusted for age, sex, study center, and the first 10 genetic principal components of ancestry within each cohort [18].

### IGAP clinically diagnosed AD GWAS dataset

The AD GWAS dataset is from the International Genomics of Alzheimer’s Project (IGAP), which is the large-scale meta-analysis of GWAS using clinically diagnosed AD (NINCDS-ADRDA criteria or DSM-IV guidelines) in individuals of European ancestry [20, 27]. The IGAP stage 1 is based on a meta-analysis of 46 GWAS datasets including 21,982 AD and 41,944 cognitively normal controls of European descent from four AD consortia including Alzheimer Disease Genetics Consortium, Cohorts for Heart and Aging Research in Genomic Epidemiology Consortium (CHARGE), The European Alzheimer’s Disease Initiative (EADI), and Genetic and Environmental Risk in AD/Defining Genetic, Polygenic and Environmental Risk for Alzheimer’s Disease Consortium (GERAD/PERADES) [20]. This AD GWAS has adjusted for age, sex, and population substructure [20]. Table 1 provides the demographic profiles about the IGAP AD GWAS dataset, as provided in the original study [20].

**Table 1:** Demographic profiles about the selected AD and AD proxy phenotype GWAS datasets

GWAS datasets	AD or AD proxy			Controls or proxy controls		
	*N*	% female	Mean AAO (s.d)	*N*	% female	Mean AAE (s.d)
IGAP ADGC	14,428	59.3	71.1 (17.3)	14,562	59.3	76.2 (9.9)
IGAP CHARGE	2137	67.3	82.6 (12)	13,474	55.8	76.7 (8.2)
IGAP EADI	2240	65	75.4 (9.1)	6631	60.6	78.9 (7.0)
IGAP GERAD	3177	64	73.0 (0.2)	7277	51.8	51.0 (0.1)
UK Biobank AD proxy	42,034	65.9	-	272,244	-	-
Maternal AD group from UK Biobank	27,696	100	-	260,980	-	-
Paternal AD group from UK Biobank	14,338	0	-	245,941	-	-

*AD* Alzheimer’s disease, *AAO* age at onset, *AAE* age at examination

### UK Biobank AD proxy phenotype GWAS datasets

The AD proxy phenotype GWAS datasets are from the large-scale GWAS of AD family history in 314,278 participants from UK Biobank [19]. UK Biobank included 502,536 community-dwelling individuals aged 37–73 years recruited in the UK between 2006 and 2010 [28]. The proportion of women was 56% and the average age was 56 (*SD* 8) in both women and men [28]. In the UK Biobank AD GWAS dataset, the AD status was assessed by self-report [19]. All participants were asked to answer the question: Has/did your father or mother ever suffer from Alzheimer’s disease/dementia? This GWAS excluded participants whose parents were aged less than 60 years, dead before reaching age 60 years, or without age information [19]. Finally, 314,278 participants provided the AD information about at least one parent and were further divided into 27,696 maternal cases and 14,338 paternal cases [19].

Meanwhile, a subgroup analysis of the UK Biobank AD proxy phenotype was conducted in maternal AD and paternal AD including 27,696 cases of maternal AD with 260,980 controls and 14,338 cases of paternal AD with 245,941 controls, as provided in Table 1 [19]. In the single maternal or paternal AD GWAS, the key covariates adjusted for including age of parent at death or at time of the offspring’s self-report, assessment center, genotype batch, array, and 40 genetic principal components [19]. Until now, the genetic correlation between clinically diagnosed AD and AD proxy phenotype has been investigated [29]. Jansen and colleagues found a strong genetic correlation of 0.81 (*SE* = 0.185) between clinically diagnosed AD status and AD proxy phenotype in UK Biobank [29]. Marioni and colleagues also identified a high genetic correlation of maternal AD (*rg* = 0.91 and *SE* = 0.24) and paternal AD (*rg* = 0.66 and *SE* = 0.39) with clinically diagnosed AD status [19].

### Cognitive performance GWAS dataset

We selected the cognitive performance GWAS dataset from Social Science Genetic Association Consortium (SSGAC) (*N* = 257,841) [21]. This is a meta-analysis of two large-scale cohorts including general cognitive ability in COGENT (Cognitive Genomics Consortium) (*N =* 35,298) and cognitive performance in UK Biobank (*N =* 222,543) [21]. The phenotype measures used in both cohorts are provided in supplementary Table 1.

### MR analysis

We selected the inverse-variance weighted (IVW) as the main MR analysis method to combine the variant-specific Wald estimators by taking the inverse of their approximate variances as the corresponding weights [25]. Meanwhile, we selected the weighted median, MR-Egger, MR pleiotropy residual sum and outlier (MR-PRESSO), and leave-one-out permutation analysis as the sensitivity analysis methods [22, 25]. The weighted median could produce consistent estimates even 50% of selected genetic variants are valid instruments [22–26]. MR-Egger is based on the same regression model with IVW, but allows and accounts for the potential pleiotropy using the MR-Egger intercept test [22–26]. If the selected genetic variants are not pleiotropic, then the MR-Egger intercept term should tend to zero as the sample size increases [25]. The MR-PRESSO global test could identify the horizontal pleiotropic outliers [22]. Importantly, MR-PRESSO could correct for the horizontal pleiotropy via outlier removal (the MR-PRESSO outlier test) [22]. The leave-one-out permutation analysis sequentially removes each genetic variant from the MR analysis and evaluates the influence of a single genetic variant on the over MR estimate [22–26].

Meanwhile, we conducted a heterogeneity test with Cochran’s *Q* statistic (together with the *I*^2^ statistic) from IVW, which could provide evidence of heterogeneity due to pleiotropy or other causes [30]. Cochran’s *Q* statistic approximately follows a *x*^2^ distribution with *k*-1 degrees of freedom (*k* stands for the number of genetic variants) [31]. $$ {I}^2=\raisebox{1ex}{$\left(Q-\left(k-1\right)\right)$}\!\left/ \!\raisebox{-1ex}{$Q$}\right.\times 100\% $$ ranges from 0 to 100%, and 0–25%, 25–50%, 50–75% and 75–100% corresponding to low, moderate, large, and extreme heterogeneity, respectively [31].

In addition to the heterogeneity test, we also performed a pleiotropy analysis using the manual methods. First, we checked the pleiotropic association of vitamin C genetic variants or their proxies using PhenoScanner [32]. PhenoScanner is a curated database holding publicly available results from large-scale GWAS [32]. The database currently contains over 65 billion associations and over 150 million unique genetic variants [32]. Hence, PhenoScanner is a useful tool for searching human genotype–phenotype associations [32]. Second, we inquired the pleiotropy analysis results from Zheng and colleagues, as they conducted a MR analysis to evaluate the causal association between plasma vitamin C and type 2 diabetes [18]. Zheng and colleagues constructed a vitamin C-raising genetic score using the 11 plasma vitamin C genetic variants in the EPIC-InterAct study and examined its association with anthropometric, lifestyle, dietary factors, and lipid biomarkers [18]. Meanwhile, Zheng and colleagues evaluated the association of 11 plasma vitamin C genetic variants with 174 blood metabolites using 174 GWAS summary statistics [18, 33].

The odds ratio (*OR*) and 95% confidence interval (*CI*) for AD or AD proxy phenotype, as well as the effect size (beta) and *95% CI* for cognitive performance, correspond to 1 standard deviation (*SD*) in vitamin C levels (1 *SD* = 20.2 μmol/l from the EPIC-Norfolk study) [18]. All the statistical tests were completed using three R Packages including “meta: General Package for Meta-Analysis,” “MendelianRandomization,” and “MR-PRESSO,” respectively [11]. The significance threshold is *P* < 0.05.

### Power analysis

We first calculated the proportion of plasma vitamin C variance *R*^2^ explained by the selected genetic variants using two formulas. The first formula was

$$ {R}^2=\sum \limits_{i=1}^K\frac{\beta_i^2}{\beta_i^2+{2}^{\ast }{N}^{\ast } se{\left({\beta}_i\right)}^2} $$

where *β*_*i*_ is the effect size for *SNP*_*i*_, *se*(*β*_i_) is the standard error for *SNP*_*i*_, *N* is the sample size for *SNP*_*i*_, and *K* is the number of the selected genetic variants [34]. The second formula was

$$ {R}^2=\sum \limits_{i=1}^k2\ast MAF\ast \left(1-\mathrm{MAF}\right)\ast {\beta}_i^2 $$

where *β*_*i*_ is the effect size for *SNP*_*i*_, *MAF* is the minor allele frequency for *SNP*_*i*_, and *K* is the number of the selected genetic variants [35]. We further calculated the statistical power using the Web-based tool mRnd and a two-sided type-I error rate *α* of 0.05 [36].

## Results

### Vitamin C genetic variants and their association with AD, AD proxy phenotype, and cognitive performance

We selected 11 genetic variants with a genome-wide significant level (*P* < 5.00E−08) as the potential instrumental variables from the recent vitamin C GWAS dataset [18]. These vitamin C genetic variants are successfully identified around 11 loci on chromosome 1 (*RER1*), 2 (*SLC23A3*), 5 (*SLC23A1* and *RGS14*), 6 (*GSTA5*), 11 (*FADS1*), 12 (*SNRPF* and *CHPT1*), 14 (*AKT1*), 16 (*MAF*), and 17 (*BCAS3*) [18]. On chromosome 5, the *SLC23A1* rs33972313 and *RGS14* rs10051765 are 38084490 bps away from each other. On chromosome 12, the *SNRPF* rs117885456 and *CHPT1* rs2559850 are 5844348 bps away from each other. Hence, all these 11 genetic variants are completely independent and not in linkage disequilibrium with each other (*r*^2^ < 0.01 and *D*’ < 0.01). All these selected 11 genetic variants could explain 1.79% and 1.84% variance of plasma vitamin C levels, as provided in Table 2. The summary results about the effect of each genetic variant on vitamin C levels and the standard errors are also provided in Table 2.

**Table 2:** Main characteristics of 11 selected plasma vitamin C genetic variants

SNP	Chromosome	Position (GRCh37)	EA	NEA	EAF	Beta	*SE*	*P* value	Gene	*R*^*2*a^	*R*^*2*b^
rs6693447	1	2330190	T	G	0.551	0.039	0.006	6.25E−10	*RER1*	0.08%	0.08%
rs13028225	2	220031255	T	C	0.857	0.102	0.009	2.38E−30	*SLC23A3*	0.25%	0.26%
rs33972313	5	138715502	C	T	0.968	0.36	0.018	4.61E−90	*SLC23A1*	0.76%	0.80%
rs10051765	5	176799992	C	T	0.342	0.039	0.007	3.64E−09	*RGS14*	0.06%	0.07%
rs7740812	6	52725787	G	A	0.594	0.038	0.006	1.88E−09	*GSTA5*	0.08%	0.07%
rs174547	11	61570783	C	T	0.328	0.036	0.007	3.84E−08	*FADS1*	0.05%	0.06%
rs117885456	12	96249111	A	G	0.087	0.078	0.012	1.70E−11	*SNRPF*	0.08%	0.10%
rs2559850	12	102093459	A	G	0.598	0.058	0.006	6.30E−20	*CHPT1*	0.18%	0.16%
rs10136000	14	105253581	A	G	0.283	0.04	0.007	1.33E−08	*AKT1*	0.06%	0.06%
rs56738967	16	79740541	C	G	0.321	0.041	0.007	7.62E−10	*MAF*	0.07%	0.07%
rs9895661	17	59456589	T	C	0.817	0.063	0.008	1.05E−14	*BCAS3*	0.12%	0.12%

*SNP* single-nucleotide polymorphism, *EA* effect allele, *NEA* non-effect allele, *EAF* effect allele frequency, *SE* standard error. Beta is the regression coefficient based on the vitamin C raising allele (effect allele); *R*^2^, the proportion of vitamin C variance explained by the selected genetic variants; ^a^ and ^b^, the proportion of plasma vitamin C variance *R*^2^ explained by the selected genetic variants was calculated using the first formula and the second formula

We further extracted the corresponding summary statistics in the IGAP AD GWAS dataset, UK Biobank AD proxy phenotype GWAS datasets, and cognitive performance GWAS dataset using the 11 vitamin C genetic variants, respectively, as provided in Table 3. It is noted that rs56738967 (C/G, C with the minor allele frequency (*MAF*) = 0.321) is an ambiguous palindromic variant (i.e., with alleles either A/T or C/G). Hence, we selected its proxy rs17689159 (C/T, C with the *MAF* = 0.29), which showed high linkage disequilibrium with rs56738967 (*r*^2^ = 1 and *D*’ = 1) using the HaploReg v4.1 based on the linkage disequilibrium information in 1000 Genomes Project (CEU) [37]. Only the *SLC23A1* rs33972313 variant was significantly associated with AD proxy phenotype in UK Biobank GWAS datasets (*P* = 4.71E−05 for AD proxy phenotype in maternal AD and *P* = 1.14E−03 for AD proxy phenotype in both maternal AD and paternal AD) using the adjusted significance threshold *P* < 0.05/11 = 4.55E−03.

**Table 3:** GWAS summary statistics corresponding to 11 vitamin C genetic variants in IGAP and UK Biobank

SNP	Plasma vitamin C GWAS			AD GWAS from IGAP		AD proxy GWAS from UK Biobank		Maternal AD group from UK Biobank		Paternal AD group from UK Biobank		Cognitive performance GWAS	
	EA	NEA	EAF	Beta	6. *SE*	Beta	7. *SE*	Beta	*SE*	Beta	*SE*	Beta	*SE*
rs10051765	C	T	0.342	−0.0126	0.016	−0.019	0.01	−0.009	0.012	−0.036	0.017	0.00272	0.00305
rs10136000	A	G	0.283	−0.0368	0.018	0.005	0.01	−0.006	0.012	0.025	0.017	−0.00183	0.00320
rs117885456	A	G	0.087	−0.0407	0.032	−0.004	0.01	−0.004	0.012	−0.005	0.017	0.00227	0.00497
rs13028225	T	C	0.857	0.0071	0.0208	0.012	0.01	0.006	0.012	0.023	0.017	0.00557	0.00412
rs174547	C	T	0.328	−0.012	0.0151	−0.002	0.01	−0.014	0.012	0.021	0.017	0.01375	0.00300
rs2559850	A	G	0.598	−0.012	0.015	−0.011	0.01	−0.014	0.012	−0.005	0.017	−0.00145	0.00290
rs33972313	C	T	0.968	0.0144	0.0428	−0.032	0.01	−0.05	0.012	0.001	0.017	−0.00561	0.00784
rs56738967^a^	C	G	0.321	0.0154	0.0166	0.006	0.01	−0.003	0.012	0.023	0.017	0.00322	0.00307
rs6693447	T	G	0.551	−0.0025	0.0148	0.01	0.01	0.007	0.012	0.015	0.017	0.00003	0.00288
rs7740812	G	A	0.594	−0.0137	0.0145	0.008	0.01	0.012	0.012	0.002	0.017	−0.00565	0.00293
rs9895661	T	C	0.817	−0.0024	0.0192	−0.014	0.01	−0.008	0.013	−0.025	0.018	−0.00288	0.00382

*SNP* single-nucleotide polymorphism, *EA* effect allele, *NEA* non-effect allele, *EAF* effect allele frequency, *SE* standard error. Beta is the regression coefficient based on the vitamin C raising allele (effect allele); ^a^we selected the rs17689159 variant (C/T, C with the *MAF* = 0.29) in high linkage disequilibrium with rs56738967 (*r*^2^ = 1 and *D*’ = 1)

### MR analysis

We first conducted a MR analysis using 11 plasma vitamin C genetic variants. In the IGAP AD GWAS dataset and the cognitive performance GWAS dataset, we did not observe any significant causal effects of plasma vitamin C levels on the risk of AD and the cognitive performance, as provided in Table 4. Interestingly, the MR analysis in the UK Biobank GWAS dataset showed that per 1 *SD* increase in vitamin C levels (about 20.2 μmol/l) was significantly associated with the reduced risk of AD proxy phenotype (*OR* = 0.93, *95% CI* 0.88–0.98, *P* = 7.00E−03) using IVW method. Importantly, three additional sensitivity analysis methods further supported the suggestive or significant association of genetically increased vitamin C levels with the reduced risk of AD proxy phenotype (Table 4). These estimates were consistent with the IVW estimate in terms of direction and magnitude including weighted median (*OR* = 0.92, *95% CI* 0.87–0.97, *P* = 2.00E−03), MR-Egger (*OR* = 0.91, *95% CI* 0.85–0.98, *P* = 9.00E−03), and MR-PRESSO (*OR* = 0.93, *95% CI* 0.88–0.98, *P* = 2.30E−02).

**Table 4:** MR analysis of the causal association of plasma vitamin C levels with AD, AD proxy phenotype, and cognitive performance using 11 genetic variants including the rs174547 variant

GWAS dataset	Method	*OR*/beta^a^	*95% CI*	*P* value
IGAP AD	IVW	0.93	0.80−1.09	3.85E−01
	Weighted median	1.01	0.83−1.23	9.25E−01
	MR-Egger	1.09	0.85−1.40	4.90E−01
	MR-PRESSO	0.93	0.81−1.08	3.80E−01
UK Biobank AD proxy	IVW	0.93	0.88−0.98	**7.00E−03**
	Weighted median	0.92	0.87−0.97	**2.00E−03**
	MR-Egger	0.91	0.85−0.98	**9.00E−03**
	MR-PRESSO	0.93	0.88−0.98	**2.30E−02**
Maternal AD group from UK Biobank	IVW	0.89	0.84−0.94	**7.29E−05**
	Weighted median	0.87	0.82−0.93	**3.30E−05**
	MR-Egger	0.87	0.80−0.94	**1.00E−03**
	MR-PRESSO	0.89	0.85−0.93	**6.65E−04**
Paternal AD group from UK Biobank	IVW	1.02	0.92−1.12	7.59E−01
	Weighted median	1.00	0.92−1.10	9.25E−01
	MR-Egger	0.99	0.86−1.14	9.08E−01
	MR-PRESSO	1.02	0.92−1.12	7.66E−01
Cognitive performance	IVW	0.007	[−0.043, 0.057]	0.775
	Weighted median	−0.012	[−0.050, 0.026]	0.546
	MR-Egger	−0.019	[−0.100, 0.061]	0.638
	MR-PRESSO	0.007	[−0.043, 0.057]	0.781

*OR* odds ratio, *CI* confidence interval, *IVW* inverse-variance weighted, *IGAP* International Genomics of Alzheimer’s Project, *MR-PRESSO* Mendelian randomization pleiotropy residual sum and outlier; the significance of suggestive association between vitamin C levels and AD was at *P* < 0.05; the significance of statistically significant association between vitamin C levels and AD was at Bonferroni-corrected significance *P* < 0.05/4 = 0.0125. ^a^OR for AD and AD proxy phenotype, and beta for cognitive performance

**Fig. 2:**
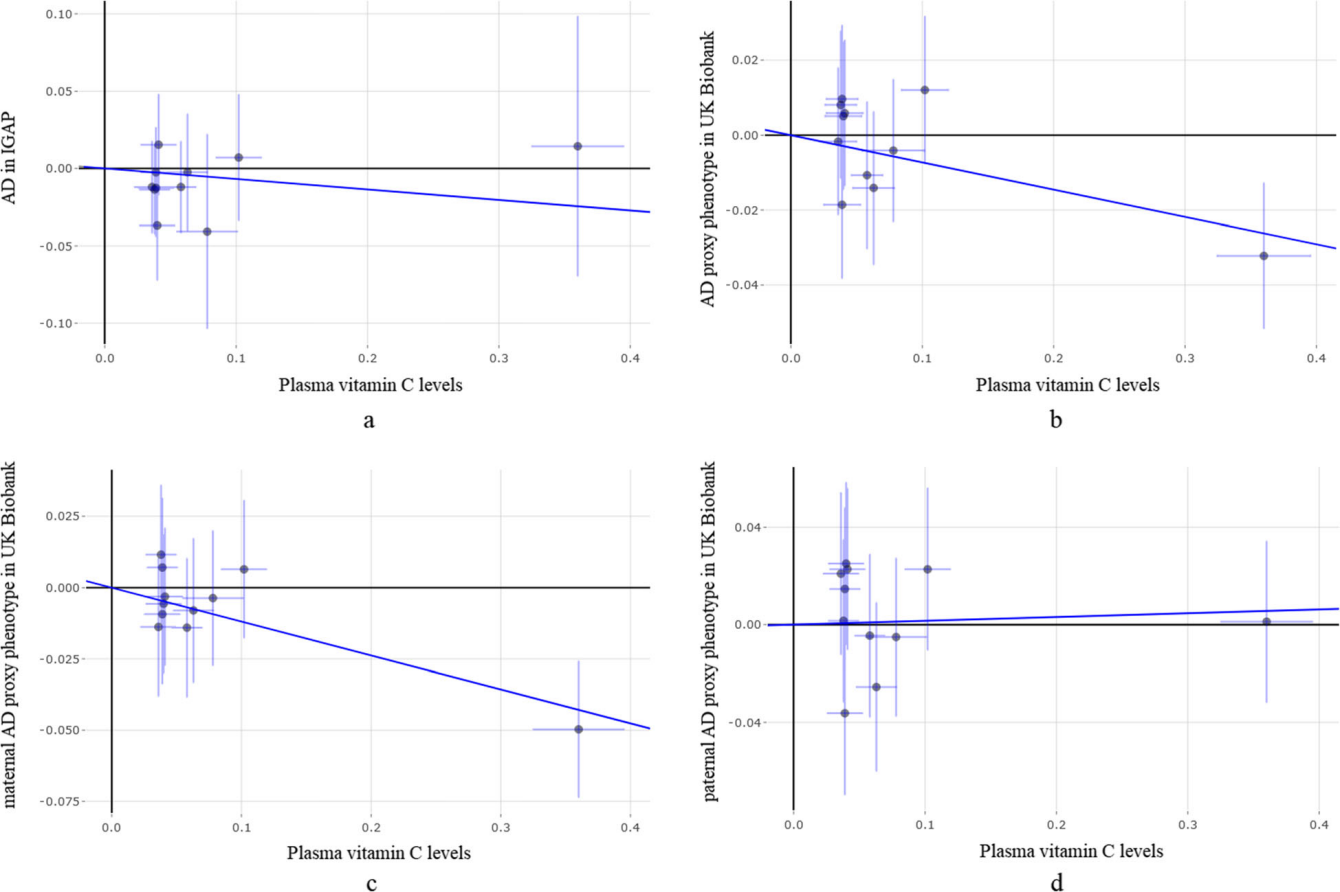
Individual MR estimates about the causal effect of plasma vitamin C levels on AD risk in IGAP and UK Biobank GWAS datasets. The *x*-axis shows the single-nucleotide polymorphism (SNP) effect, and standard error, on plasma vitamin C levels for each of the 11 SNPs, and the *y*-axis shows the SNP effect and standard error on AD. The regression line for the inverse-variance weighted method is shown. **a** IGAP AD GWAS dataset; **b** UK Biobank AD GWAS dataset; **c** UK Biobank female AD GWAS dataset; **d** UK Biobank male AD GWAS dataset

We further conducted a subgroup MR analysis in UK Biobank GWAS datasets. Interestingly, the results indicated that genetically increased vitamin C levels could significantly reduce the risk of AD proxy phenotype in the maternal AD group, but not in the paternal AD group (Table 4). IWV showed that per 1 *SD* increase in vitamin C levels (about 20.2 μmol/l) could reduce 11% risk of AD proxy phenotype in the maternal AD group with *OR* = 0.89, *95% CI* 0.84–0.94, and *P* = 7.29E−05. Importantly, three additional sensitivity analysis methods including weighted median, MR-Egger, and MR-PRESSO further supported this finding in the maternal AD group (Table 4). Figure 2 shows the individual MR estimates about the causal effect of vitamin C levels on the risk of AD in IGAP and the risk of AD proxy phenotype in UK Biobank GWAS datasets using the IVW method, respectively.

Using the three statistical methods, we did not identify any significant pleiotropic variant among the selected 11 vitamin C genetic variants in the IGAP AD and UK Biobank AD proxy phenotype GWAS datasets. In brief, the MR-Egger intercept test showed no significant pleiotropy in the IGAP AD GWAS dataset (intercept = −0.013 and *P* = 0.116) and UK Biobank AD proxy phenotype GWAS datasets (intercept = 0.004 and *P* varying from 0.354 to 0.616). The MR-PRESSO global test identified no horizontal pleiotropic outliers with *P* varying from 0.1785 to 0.5045. The heterogeneity test from IVW showed Cochran’s *Q* statistics *P* varying from 0.1409 to 0.7701 and *I*^2^ varying from 0.0 to 32.3%. In the cognitive performance GWAS dataset, the MR-PRESSO global test identified the rs174547 variant to be a pleiotropic outlier. Importantly, the heterogeneity test of 11 genetic variants from IVW showed Cochran’s *Q* statistics *P =* 8E−04 and *I*^2^ = 66.7%. Meanwhile, the heterogeneity test of 10 genetic variants excluding rs174547 from IVW showed Cochran’s *Q* statistics *P=*0.4184 and *I*^2^ = 2.2%. Hence, rs174547 may be a pleiotropic variant in the cognitive performance GWAS dataset. More detailed pleiotropy analysis results are provided in Table 5.

**Table 5:** Pleiotropy analysis of 11 selected plasma vitamin C genetic variants

GWAS dataset	MR-Egger intercept			MR-PRESSO global test	Heterogeneity test from IVW		
	Intercept	8. *95% CI*	*P* value	*P* value	*I*^*2*^	*95% CI*	*Q* statistics *P* value
IGAP	−0.013	[−0.030, 0.003]	0.116	0.5045	0.0%	[0.0%, 55.6%]	0.5357
Maternal AD group from UK Biobank	0.004	[−0.006, 0.014]	0.416	0.4305	0.0%	[0.0%, 39.0%]	0.7701
Paternal AD group from UK Biobank	0.004	[−0.013, 0.021]	0.616	0.297	32.3%	[0.0%, 66.7%]	0.1409
UK Biobank AD proxy	0.004	[−0.005, 0.013]	0.354	0.1785	17.7%	[0.0%, 57.9%]	0.2748
Cognitive performance	0.002	[−0.003, 0.008]	0.404	0.0035	66.7%	[37.2% 82.4%]	8E-04

The significance threshold is *P* < 0.05

Using the manual methods in PhenoScanner, we found that the rs174547 variant was significantly associated with a large number of lipid metabolism and lipid biomarkers at the genome-wide significance level (*P* < 5.00E−08), as provided in supplementary Table 2 [32]. Meanwhile, Zheng and colleagues also found rs174547 to be significantly associated with glycerophospholipids or sphingolipids [33]. Until now, the causal association between cholesterol and AD has been established, especially the high-density lipoprotein (HDL) cholesterol [38, 39], low-density lipoprotein (LDL) cholesterol [40], and total cholesterol [40]. Hence, rs174547 may be a pleiotropic variant. We further conducted a MR analysis using 10 plasma vitamin C variants excluding the rs174547 variant and found the similar findings as the MR analysis including the rs174547 variant, as provided in Table 6.

**Table 6:** MR analysis of the causal association of plasma vitamin C levels with AD, AD proxy phenotype, and cognitive performance using 10 genetic variants excluding the rs174547 variant

GWAS dataset	Method	OR/beta^a^	*95% CI*	*P* value
IGAP AD	IVW	0.94	0.81–1.10	4.64E−01
	Weighted median	1.02	0.83–1.24	8.83E−01
	MR-Egger	1.09	0.85–1.40	5.07E−01
	MR-PRESSO	0.94	0.81–1.10	4.71E−01
UK Biobank AD proxy	IVW	0.93	0.88–0.98	**1.30E−02**
	Weighted median	0.92	0.87–0.97	**2.00E−03**
	MR-Egger	0.91	0.84–0.98	**1.70E−02**
	MR-PRESSO	0.93	0.88–0.98	**3.41E−02**
Maternal AD group from UK Biobank	IVW	0.89	0.84–0.94	**8.38E−05**
	Weighted median	0.87	0.82–0.93	**3.30E−05**
	MR-Egger	0.86	0.80–0.93	**2.54E−04**
	MR-PRESSO	0.89	0.85–0.93	**1.05E−03**
Paternal AD group from UK Biobank	IVW	1.01	0.91–1.12	8.25E−01
	Weighted median	1.00	0.92–1.10	9.38E−01
	MR-Egger	1.00	0.87–1.15	9.95E−01
	MR-PRESSO	1.01	0.91–1.12	8.30E−01
Cognitive performance	IVW	−0.005	[−0.034, 0.025]	0.754
	Weighted median	−0.013	[−0.051, 0.025]	0.510
	MR-Egger	−0.004	[−0.054, 0.045]	0.865
	MR-PRESSO	−0.005	[−0.034, 0.025]	0.761

*OR* odds ratio, *CI* confidence interval, *IVW* inverse-variance weighted, *IGAP* International Genomics of Alzheimer’s Project, *MR-PRESSO* Mendelian randomization pleiotropy residual sum and outlier; the significance of suggestive association between vitamin C levels and AD was at *P* < 0.05; the significance of statistically significant association between vitamin C levels and AD was at Bonferroni-corrected significance *P* < 0.05/4 = 0.0125. ^a^*OR* for AD and AD proxy phenotype, and beta for cognitive performance

**Fig. 3:**
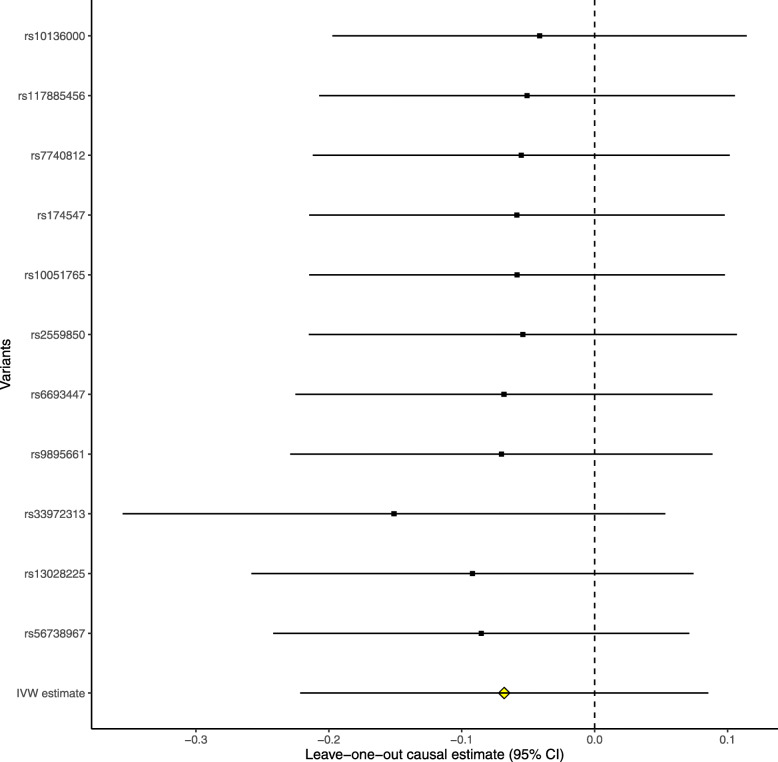
Leave-one-out permutation analysis of the causal association between plasma vitamin C levels and AD in the IGAP GWAS dataset using the IVW method

**Fig. 4:**
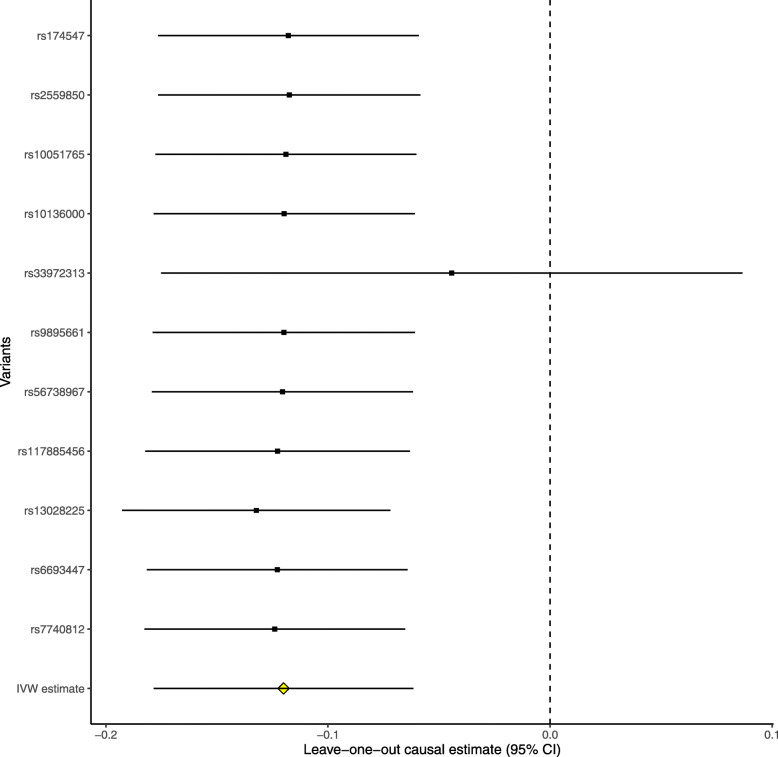
Leave-one-out permutation analysis of the causal association between plasma vitamin C levels and the AD proxy phenotype in the UK Biobank GWAS dataset using the IVW method

**Fig. 5:**
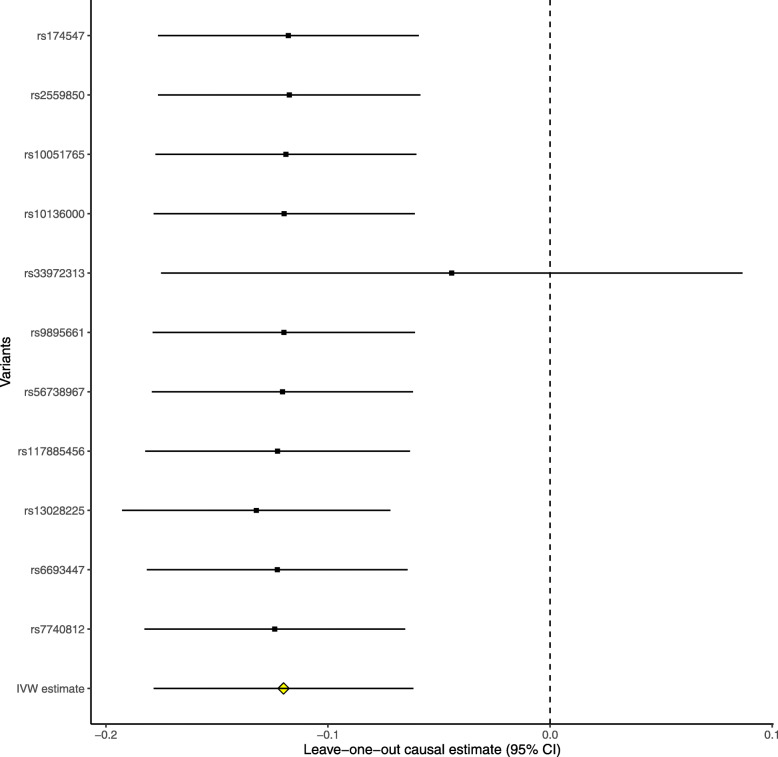
Leave-one-out permutation analysis of the causal association between plasma vitamin C levels and the AD proxy phenotype in the UK Biobank maternal AD GWAS dataset using the IVW method

**Fig. 6:**
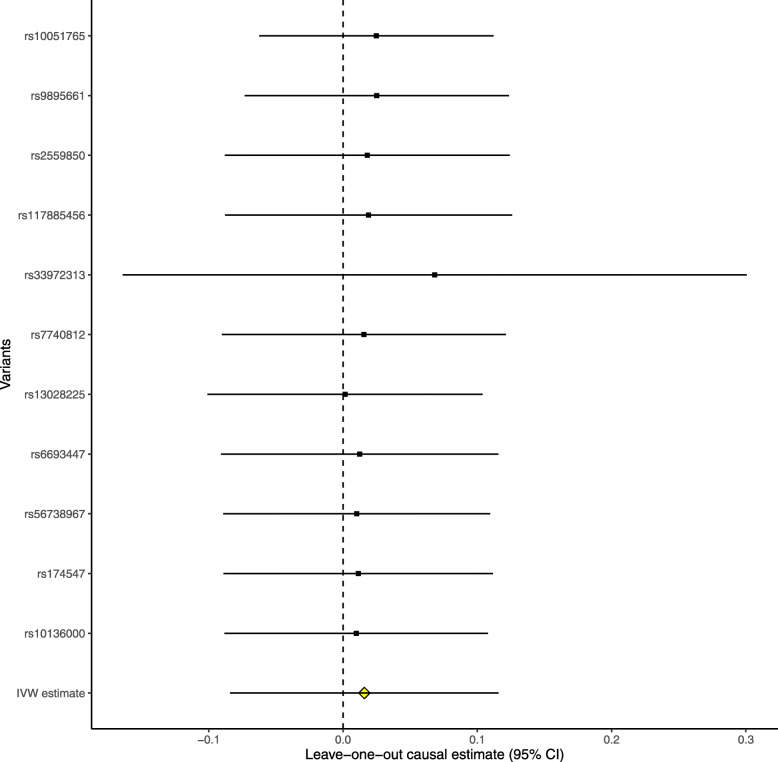
Leave-one-out permutation analysis of the causal association between plasma vitamin C levels and the AD proxy phenotype in the UK Biobank paternal AD GWAS dataset using the IVW method

**Fig. 7:**
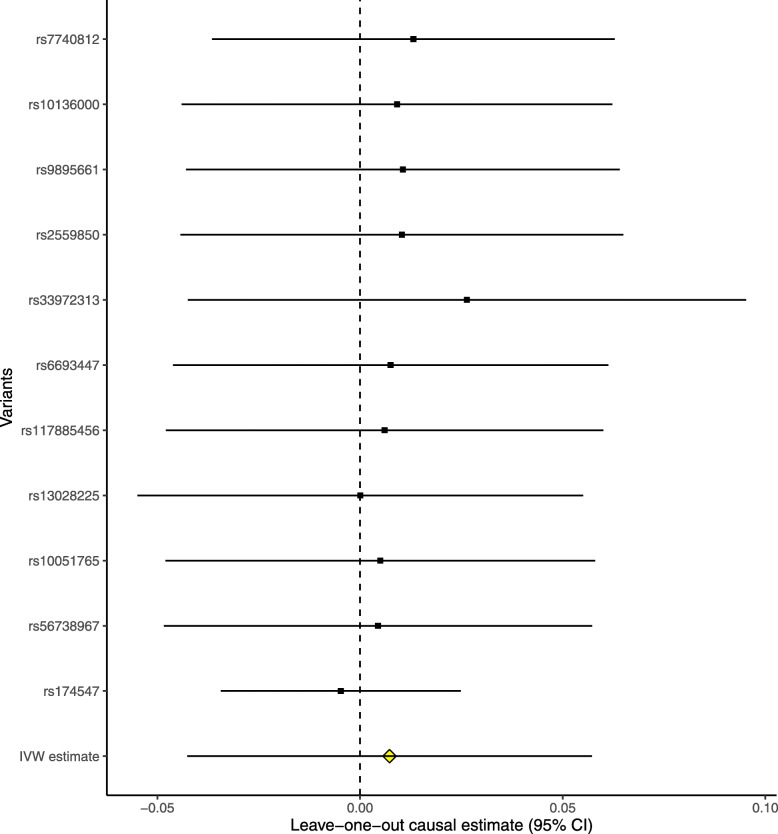
Leave-one-out permutation analysis of the causal association between plasma vitamin C levels and cognitive performance in the cognitive performance GWAS dataset using the IVW method

The leave-one-out permutation further showed that only the *SLC23A1* rs33972313 variant largely changed the precision of the overall MR estimates in the IGAP AD GWAS dataset (Fig. 3), UK Biobank AD proxy phenotype GWAS dataset (Fig. 4), UK Biobank maternal AD and paternal AD GWAS datasets (Figs. 5 and 6), and cognitive performance GWAS dataset (Fig. 7). Importantly, MR analysis excluding the rs33972313 variant indicates no significant causal association between plasma vitamin C levels and the risk of AD proxy phenotype, as provided in Figs. 4, 5, and 6. Hence, the *SLC23A1* rs33972313 variant may have driven the causal association in UK Biobank GWAS datasets.

### Power analysis

All these selected 11 genetic variants could explain 1.79–1.84% variance of plasma vitamin C levels. In the IGAP GWAS dataset, our MR study had 80% power to detect an *OR* of 0.84 or lower per *SD* increase in vitamin C levels (about 20.2 μmol/l) for AD. In UK Biobank GWAS datasets, our MR study had 80% power to detect *OR* of 0.90, 0.87, and 0.83 or lower per *SD* increase in vitamin C levels (about 20.2 μmol/l) for AD proxy phenotype, maternal AD, and paternal AD, respectively. Interestingly, the power is 80% to detect the causal association between increased vitamin C levels and reduced risk of maternal AD with *OR* = 0.87, as reported in the UK Biobank maternal AD GWAS dataset. These detective *OR*s are comparable with a previous observational study, which reported that per 1 *SD* increase in vitamin C intake levels could reduce the risk of AD with rate ratio 0.82 [6].

## Discussion

Until now, observational studies have explored the impact of vitamin C intake (both diet and supplements) on AD risk [6–10]. Some observational studies found that high vitamin C levels could reduce the risk of AD. However, others reported ambiguous findings [6–10]. In order to develop effective therapies or prevention, the causal link between vitamin C levels and AD should be established. Hence, it is necessary to improve the causal inference through other study designs and overcome the methodological limitations of observational studies [11]. Hence, we performed an updated two-sample MR study using multiple large-scale GWAS datasets from plasma vitamin C (*N* = 52,018), IGAP clinically diagnosed AD (*N* = 63,926), UK Biobank AD proxy phenotype (*N* = 314,278), UK Biobank maternal AD (27,696 cases of maternal AD and 260,980 controls) and paternal AD (14,338 cases of paternal AD and 245,941 controls), and cognitive performance (*N*= 257,841).

In IGAP, we did not identify any significant causal association between plasma vitamin C levels and the risk of AD. Meanwhile, we did not observe any significant causal effect of plasma vitamin C levels on cognitive performance. Hence, there may be no causal association between plasma vitamin C levels and the risk of AD in people of European descent. In UK Biobank, we found that per 1 *SD* increase in plasma vitamin C levels (about 20.2 μmol/l) was significantly associated with the reduced risk of AD proxy phenotype using four MR methods including IVW, weighted median, MR-Egger, and MR-PRESSO. A subgroup analysis in the UK Biobank maternal AD group further supported these above findings, but not in the paternal AD group. These findings may indicate the gender differences in the causal association between plasma vitamin C levels and the risk of AD.

We consider that the inconsistent findings in clinically diagnosed AD and AD proxy phenotype may be caused by phenotypic heterogeneity. In UK Biobank, the proxy phenotype for AD and control was assessed via self-report [19]. Participants were asked to report “Has/did your father or mother ever suffer from Alzheimer’s disease/dementia?” [19]. However, not all UK Biobank participants could exactly discriminate AD from other dementia sub-types, because of different presentations and genetic architectures [19, 41]. Hence, the self-report parental AD status may not exactly reflect the clinically diagnosed AD status and further cause the phenotypic heterogeneity [19, 41].

Our MR findings in IGAP are comparable to those from Williams and colleagues [17]. Williams and colleagues selected one vitamin C genetic variant rs33972313 as the instrument, which was identified in 15,087 participants [42], and selected the AD GWAS including 54,162 individuals of European ancestry (17,008 AD cases and 37,154 controls) [27]. They found no causal association between circulating vitamin C levels and the risk of AD [17]. Here, we selected 11 vitamin C genetic variants including rs33972313 and other 10 novel genetic variants as the instrumental variables, which were identified in 52,018 individuals of European ancestry [18], and the large-scale AD GWAS datasets from IGAP (*n* = 63,926, 21,982 AD and 41,944 controls) [20].

The leave-one-out permutation analysis indicated that the *SLC23A1* rs33972313 variant may have driven the causal association in UK Biobank GWAS datasets. *SLC23A1* rs33972313 is the first genetic variant associated with plasma vitamin C levels, which is identified by a candidate gene approach [42]. Importantly, the *SLC23A1* rs33972313 variant was further reported to be the strongest signal associated with plasma vitamin C levels by the GWAS method [18]. *SLC23A1* encodes solute carrier family 23 member 1 and plays an important role in the uptake of vitamin C into target tissues [43].

Until now, the observational studies supported the gender differences in AD [44]. The AD prevalence was 1.7-fold higher in females compared to males [19]. Our findings show that high vitamin C levels may improve AD in females. Interestingly, evidence from observational studies supported our findings. A pilot cross-sectional study in healthy adults showed gender differences in plasma vitamin C concentrations and cognitive function [45]. Compared with males with inadequate plasma vitamin C levels, females with adequate vitamin C concentrations were more compromised on tasks involving psychomotor performance/motor speed and exhibited higher performance on tasks involving recall, recognition, attention, and focus [45]. The Rotterdam Study consisted of 5395 participants with the mean age 67.7 years (59% female, *n* = 3183) [6]. This study identified a weak association between per 1 *SD* increase in vitamin C levels and reduced AD risk with *RR* = 0.82, *95% CI* 0.68–0.99, and *P* = 0.04 [6]. The MAP analyzed 925 participants with average age 81 years, 75% female, and 98% White [10]. This study showed that the highest quartile intakes of vitamin C (Q4) were associated with reduced AD risk compared with the lowest quartile (Q1) with *HR* = 0.64, *95% CI* 0.45–0.92, and *P* = 0.01 [10]. A recent cross-sectional study evaluated the association of plasma vitamin C concentrations with cognitive function using 80 healthy adults with mean age 60.97 years (65% female, *n* = 52) [46]. This study demonstrated that high plasma vitamin C levels were associated with improved attention, focus, working memory, decision speed, delayed and total recall, and recognition [46]. Until recently, Travica and colleagues conducted a systemic review and highlighted the gender differences in plasma and brain vitamin C levels, which may further contribute to differences in gender-associated cognitive ability [47].

Evidence shows that vitamin C supplementation using both oral administration and intravenous administration could increase the plasma vitamin C levels [48]. The oral administration may reach a maximum level of about 70–80 μmol/L, and the intravenous administration may even reach a maximum level around 50 mmol/L [48]. Therefore, the vitamin C concentration achieved by supplementation is higher than the genetically determined per *SD* increase (20.2 μmol/l) [48]. Importantly, there is no difference in the effects of the plasma vitamin C concentrations on cognitive performance between vitamin C supplementation and dietary intake [46]. Until now, several randomized controlled trials (RCTs) have evaluated the effects of vitamin C supplementation on AD cerebrospinal fluid (CSF) biomarkers [49] and cognitive function [50, 51]. The results showed that 800 IU/day of vitamin E plus 500 mg/day of vitamin C plus 900 mg/day of α-lipoic acid did not influence CSF biomarkers [49]. Interestingly, vitamin C supplementation effectively improved attention, motivation, and reaction time and contributed to better performance on cognitive tasks requiring sustained attention [51]. Hence, our findings may have clinical and public health implications.

Our MR study may have some limitations. First, our MR analysis just reflects the findings in European ancestry. The causal association between vitamin C levels and AD risk may be different across different ancestries. Hence, our findings should be further replicated in other ancestries. Second, the IGAP and UK Biobank GWAS datasets are from the clinically diagnosed AD and self-report AD proxy phenotype, respectively [19]. Hence, there may be some differences across the different diagnostic criteria. Here, we only performed the subgroup analysis MR analysis in UK Biobank GWAS datasets [19]. However, this subgroup analysis MR analysis is not the real gender-specific MR analysis. The plasma vitamin C genetic variants are from the 52,018 individuals of European ancestry including both men and women [18]. In the original study, the AD GWAS in UK Biobank include 27,696 cases of maternal AD with 260,980 controls and 14,338 cases of paternal AD with 245,941 controls. However, these controls consist of both men and women [19]. We think that it is necessary to conduct a gender-specific MR analysis. However, the unavailable gender-specific plasma vitamin C and AD GWAS datasets limit our further analysis. Third, the plasma vitamin C GWAS dataset and four AD GWAS datasets are all from the individuals of European ancestry, as described in the “Materials and methods” section [18–20]. Hence, we could not exclude the sample overlap between the exposure and outcome data sources. However, we could not exactly check the sample overlap between the exposure and outcome data sources, as the individual information in all these GWAS datasets is not publicly available [18–20]. Fourth, only the observational studies supported the gender differences in AD and the gender differences in plasma vitamin C concentrations and cognitive function. Hence, biological evidence is required to reveal the mechanisms underlying the causal association.

## Conclusions

Our MR analysis demonstrated that there may be no causal association between plasma vitamin C levels and the risk of AD risk in people of European descent. However, our findings from the UK Biobank AD proxy phenotype may indicate the gender differences in the causal association between plasma vitamin C levels and the risk of AD.

## Supplementary Information

**Additional file 1: Supplementary Table 1.** Phenotype Descriptions and distributions in Cognitive Performance GWAS

**Additional file 2: Supplementary Table 2.** Pleiotropic association of 11 vitamin C genetic variants with human phenotypes at the genome wide significance level (*P*<5.00E-08) using PhenoScanner

## Data Availability

All relevant data are within the paper. The authors confirm that all data underlying the findings are either fully available without restriction through consortia websites or may be made available from consortia upon request. IGAP GWAS dataset: https://www.niagads.org/datasets/ng00075; UK Biobank AD GWAS dataset: https://www.ccace.ed.ac.uk/node/335; cognitive performance GWAS dataset: https://www.thessgac.org/data.

## Abbreviations

*AD*: *Alzheimer’s disease*
MR: Mendelian randomization
GWAS: Genome-wide association study
IGAP: International Genomics of Alzheimer’s Project
MR-PRESSO: MR pleiotropy residual sum and outlier
IVW: Inverse-variance weighted

## References

[1] Congdon EE, Sigurdsson EM (2018). Tau-targeting therapies for Alzheimer disease. Nat Rev Neurol.

[2] Grundman M (2000). Vitamin E and Alzheimer disease: the basis for additional clinical trials. Am J Clin Nutr.

[3] Heo JH, Hyon L, Lee KM (2013). The possible role of antioxidant vitamin C in Alzheimer’s disease treatment and prevention. Am J Alzheimers Dis Other Demen.

[4] Bitan G, Kirkitadze MD, Lomakin A, Vollers SS, Benedek GB, Teplow DB (2003). Amyloid beta -protein (Abeta) assembly: Abeta 40 and Abeta 42 oligomerize through distinct pathways. Proc Natl Acad Sci U S A.

[5] Kook SY, Lee KM, Kim Y, Cha MY, Kang S, Baik SH, Lee H, Park R, Mook-Jung I (2014). High-dose of vitamin C supplementation reduces amyloid plaque burden and ameliorates pathological changes in the brain of 5XFAD mice. Cell Death Dis.

[6] Engelhart MJ, Geerlings MI, Ruitenberg A, van Swieten JC, Hofman A, Witteman JC, Breteler MM (2002). Dietary intake of antioxidants and risk of Alzheimer disease. JAMA.

[7] Luchsinger JA, Tang MX, Shea S, Mayeux R (2003). Antioxidant vitamin intake and risk of Alzheimer disease. Arch Neurol.

[8] Zandi PP, Anthony JC, Khachaturian AS, Stone SV, Gustafson D, Tschanz JT, Norton MC, Welsh-Bohmer KA, Breitner JC (2004). Reduced risk of Alzheimer disease in users of antioxidant vitamin supplements: the Cache County Study. Arch Neurol.

[9] Morris MC, Evans DA, Bienias JL, Tangney CC, Bennett DA, Aggarwal N, Wilson RS, Scherr PA (2002). Dietary intake of antioxidant nutrients and the risk of incident Alzheimer disease in a biracial community study. JAMA.

[10] Agarwal P, Holland TM, Wang Y, Bennett DA, Morris MC. Association of strawberries and anthocyanidin intake with Alzheimer’s dementia risk. Nutrients. 2019;11(12). 10.3390/nu11123060. 10.3390/nu11123060 PMC6950087 31847371

[11] Liu G, Zhao Y, Jin S, Hu Y, Wang T, Tian R, Han Z, Xu D, Jiang Q (2018). Circulating vitamin E levels and Alzheimer’s disease: a Mendelian randomization study. Neurobiol Aging.

[12] Mokry LE, Ross S, Morris JA, Manousaki D, Forgetta V, Richards JB (2016). Genetically decreased vitamin D and risk of Alzheimer disease. Neurology.

[13] Larsson SC, Traylor M, Malik R, Dichgans M, Burgess S, Markus HS (2017). Modifiable pathways in Alzheimer’s disease: Mendelian randomisation analysis. BMJ.

[14] Larsson SC, Traylor M, Markus HS. Michaelsson K, Serum parathyroid hormone, 25-hydroxyvitamin D, and risk of Alzheimer’s disease: a Mendelian randomization study. Nutrients. 2018;10(9). 10.3390/nu10091243. 10.3390/nu10091243 PMC6165184 30200567

[15] Wang L, Qiao Y, Zhang H, Zhang Y, Hua J, Jin S, Liu G (2020). Circulating vitamin D levels and Alzheimer’s disease: a Mendelian randomization study in the IGAP and UK Biobank. J Alzheimers Dis.

[16] Gagliano Taliun SA (2019). Genetic determinants of low vitamin B12 levels in Alzheimer’s disease risk. Alzheimers Dement (Amst).

[17] Williams DM, Hagg S, Pedersen NL (2019). Circulating antioxidants and Alzheimer disease prevention: a Mendelian randomization study. Am J Clin Nutr.

[18] Zheng JS, Luan J, Sofianopoulou E, Imamura F, Stewart ID, Day FR, Pietzner M, Wheeler E, Lotta LA, Gundersen TE (2021). Plasma vitamin C and type 2 diabetes: genome-wide association study and Mendelian randomization analysis in European populations. Diabetes Care.

[19] Marioni RE, Harris SE, Zhang Q, McRae AF, Hagenaars SP, Hill WD, Davies G, Ritchie CW, Gale CR, Starr JM (2018). GWAS on family history of Alzheimer’s disease. Transl Psychiatry.

[20] Kunkle BW, Grenier-Boley B, Sims R, Bis JC, Damotte V, Naj AC, Boland A, Vronskaya M, van der Lee SJ, Amlie-Wolf A (2019). Genetic meta-analysis of diagnosed Alzheimer’s disease identifies new risk loci and implicates Abeta, tau, immunity and lipid processing. Nat Genet.

[21] Lee JJ, Wedow R, Okbay A, Kong E, Maghzian O, Zacher M, Nguyen-Viet TA, Bowers P, Sidorenko J, Karlsson Linner R (2018). Gene discovery and polygenic prediction from a genome-wide association study of educational attainment in 1.1 million individuals. Nat Genet.

[22] Verbanck M, Chen CY, Neale B, Do R (2018). Detection of widespread horizontal pleiotropy in causal relationships inferred from Mendelian randomization between complex traits and diseases. Nat Genet.

[23] Bowden J, Davey Smith G, Haycock PC, Burgess S (2016). Consistent estimation in Mendelian randomization with some invalid instruments using a weighted median estimator. Genet Epidemiol.

[24] Yavorska OO, Burgess S (2017). MendelianRandomization: an R package for performing Mendelian randomization analyses using summarized data. Int J Epidemiol.

[25] Burgess S, Thompson SG (2017). Interpreting findings from Mendelian randomization using the MR-Egger method. Eur J Epidemiol.

[26] Bowden J, Davey Smith G, Burgess S (2015). Mendelian randomization with invalid instruments: effect estimation and bias detection through Egger regression. Int J Epidemiol.

[27] Lambert JC, Ibrahim-Verbaas CA, Harold D, Naj AC, Sims R, Bellenguez C, DeStafano AL, Bis JC, Beecham GW, Grenier-Boley B (2013). Meta-analysis of 74,046 individuals identifies 11 new susceptibility loci for Alzheimer’s disease. Nat Genet.

[28] Sudlow C, Gallacher J, Allen N, Beral V, Burton P, Danesh J, Downey P, Elliott P, Green J, Landray M, Liu B, Matthews P, Ong G, Pell J, Silman A, Young A, Sprosen T, Peakman T, Collins R (2015). UK biobank: an open access resource for identifying the causes of a wide range of complex diseases of middle and old age. PLoS Med.

[29] Jansen IE, Savage JE, Watanabe K, Bryois J, Williams DM, Steinberg S, Sealock J, Karlsson IK, Hagg S, Athanasiu L (2019). Genome-wide meta-analysis identifies new loci and functional pathways influencing Alzheimer’s disease risk. Nat Genet.

[30] Greco MF, Minelli C, Sheehan NA, Thompson JR (2015). Detecting pleiotropy in Mendelian randomisation studies with summary data and a continuous outcome. Stat Med.

[31] Liu G, Zhang S, Cai Z, Ma G, Zhang L, Jiang Y, Feng R, Liao M, Chen Z, Zhao B, Li K (2013). PICALM gene rs3851179 polymorphism contributes to Alzheimer’s disease in an Asian population. Neuromolecular Med.

[32] Kamat MA, Blackshaw JA, Young R, Surendran P, Burgess S, Danesh J, Butterworth AS, Staley JR (2019). PhenoScanner V2: an expanded tool for searching human genotype-phenotype associations. Bioinformatics.

[33] Lotta LA, Pietzner M, Stewart ID, Wittemans LBL, Li C, Bonelli R, Raffler J, Biggs EK, Oliver-Williams C, Auyeung VPW (2021). A cross-platform approach identifies genetic regulators of human metabolism and health. Nat Genet.

[34] Shim H, Chasman DI, Smith JD, Mora S, Ridker PM, Nickerson DA, Krauss RM, Stephens M (2015). A multivariate genome-wide association analysis of 10 LDL subfractions, and their response to statin treatment, in 1868 Caucasians. PLoS One.

[35] Locke AE, Kahali B, Berndt SI, Justice AE, Pers TH, Day FR, Powell C, Vedantam S, Buchkovich ML, Yang J (2015). Genetic studies of body mass index yield new insights for obesity biology. Nature.

[36] Brion MJ, Shakhbazov K, Visscher PM (2013). Calculating statistical power in Mendelian randomization studies. Int J Epidemiol.

[37] Ward LD, Kellis M (2012). HaploReg: a resource for exploring chromatin states, conservation, and regulatory motif alterations within sets of genetically linked variants. Nucleic Acids Res.

[38] Kjeldsen EW, Thomassen JQ, Juul Rasmussen I, Nordestgaard BG, Tybjaerg-Hansen A, Frikke-Schmidt R. Plasma HDL cholesterol and risk of dementia - observational and genetic studies. Cardiovasc Res. 2021. 10.1093/cvr/cvab164. 10.1093/cvr/cvab164 33964140

[39] Lord J, Jermy B, Green R, Wong A, Xu J, Legido-Quigley C, et al. Mendelian randomization identifies blood metabolites previously linked to midlife cognition as causal candidates in Alzheimer’s disease. Proc Natl Acad Sci U S A. 2021;118(16). 10.1073/pnas.2009808118. 10.1073/pnas.2009808118 PMC8072203 33879569

[40] Zhang Q, Xu F, Wang L, Zhang WD, Sun CQ, Deng HW (2020). Detecting potential causal relationship between multiple risk factors and Alzheimer’s disease using multivariable Mendelian randomization. Aging (Albany NY).

[41] Andrews SJ, Fulton-Howard B, Goate A (2020). Interpretation of risk loci from genome-wide association studies of Alzheimer’s disease. Lancet Neurol.

[42] Timpson NJ, Forouhi NG, Brion MJ, Harbord RM, Cook DG, Johnson P, McConnachie A, Morris RW, Rodriguez S, Luan J (2010). Genetic variation at the SLC23A1 locus is associated with circulating concentrations of L-ascorbic acid (vitamin C): evidence from 5 independent studies with >15,000 participants. Am J Clin Nutr.

[43] Shaghaghi MA, Kloss O, Eck P (2016). Genetic variation in human vitamin C transporter genes in common complex diseases. Adv Nutr.

[44] Ferretti MT, Iulita MF, Cavedo E, Chiesa PA, Schumacher Dimech A, Santuccione Chadha A, Baracchi F, Girouard H, Misoch S, Giacobini E (2018). Sex differences in Alzheimer disease - the gateway to precision medicine. Nat Rev Neurol.

[45] Travica N, Ried K, Hudson I, Sali A, Scholey A, Pipingas A (2020). Gender differences in plasma vitamin C concentrations and cognitive function: a pilot cross-sectional study in healthy adults. Curr Dev Nutr.

[46] Travica N, Ried K, Sali A, Hudson I, Scholey A, Pipingas A (2019). Plasma vitamin C concentrations and cognitive function: a cross-sectional study. Front Aging Neurosci.

[47] Travica N, Ried K, Hudson I, Sali A, Scholey A, Pipingas A (2020). The contribution of plasma and brain vitamin C on age and gender-related cognitive differences: a mini-review of the literature. Front Integr Neurosci.

[48] Lykkesfeldt J (2020). On the effect of vitamin C intake on human health: how to (mis)interprete the clinical evidence. Redox Biol.

[49] Galasko DR, Peskind E, Clark CM, Quinn JF, Ringman JM, Jicha GA, Cotman C, Cottrell B, Montine TJ, Thomas RG, Aisen P, Alzheimer's Disease Cooperative Study (2012). Antioxidants for Alzheimer disease: a randomized clinical trial with cerebrospinal fluid biomarker measures. Arch Neurol.

[50] Rutjes AW, Denton DA, Di Nisio M, Chong LY, Abraham RP, Al-Assaf AS, Anderson JL, Malik MA, Vernooij RW, Martinez G (2018). Vitamin and mineral supplementation for maintaining cognitive function in cognitively healthy people in mid and late life. Cochrane Database Syst Rev.

[51] Sim M, Hong S, Jung S, Kim JS, Goo YT, Chun WY, et al. Vitamin C supplementation promotes mental vitality in healthy young adults: results from a cross-sectional analysis and a randomized, double-blind, placebo-controlled trial. Eur J Nutr. 2021. 10.1007/s00394-021-02656-3. 10.1007/s00394-021-02656-3 PMC8783887 34476568

